# Differential expression of olfactory genes in Atlantic salmon (*Salmo salar*) during the parr–smolt transformation

**DOI:** 10.1002/ece3.5845

**Published:** 2019-11-28

**Authors:** Steffen S. Madsen, Sara S. T. Winther, Rebecca J. Bollinger, Ulrich Steiner, Martin H. Larsen

**Affiliations:** ^1^ Department of Biology University of Southern Denmark Odense Denmark; ^2^ Danish Centre for Wild Salmon Randers Denmark

**Keywords:** homing, imprinting, odorant receptors, olfaction, salmonids

## Abstract

The anadromous salmon life cycle includes two migratory events, downstream smolt migration and adult homing migration, during which they must navigate with high precision. During homing migration, olfactory cues are used for navigation in coastal and freshwater areas, and studies have suggested that the parr**–**smolt transformation has a sensitive period for imprinting. Accordingly, we hypothesized that there would be significant changes in gene expression in the olfactory epithelium specifically related to smoltification and sampled olfactory rosettes from hatchery‐reared upper growth modal juvenile Atlantic salmon at 3‐week intervals from January to June, using lower growth modal nonsmolting siblings as controls. A suite of olfactory receptors and receptor‐specific proteins involved in functional aspects of olfaction and peripheral odor memorization was analyzed by qPCR. Gene expression in juveniles was compared with mature adult salmon of the same genetic strain caught in the river Gudenaa. All mRNAs displayed significant variation over time in both modal groups. Furthermore, five receptor genes (*olfc13.1*, *olfc15.1*, *sorb*, *ora2*, and *asor1*) and four olfactory‐specific genes (*soig*, *ependymin*, *gst*, and *omp2*) were differentially regulated between modal groups, suggesting altered olfactory function during smoltification. Several genes were differentially regulated in mature salmon compared with juveniles, suggesting that homing and odor recollection involve a different set of genes than during imprinting. Thyroid hormone receptors *thrα* and *thrβ* mRNAs were elevated during smolting, suggesting increased sensitivity to thyroid hormones. Treatment of presmolts with triiodothyronine in vivo and ex vivo had, however, only subtle effects on the investigated olfactory targets, questioning the hypothesis that thyroid hormones directly regulate gene expression in the olfactory epithelium.

## Graphical abstract

Imprinting in salmon involves odor recognition. This study analyzed 17 olfactory‐related genes in Atlantic salmon on a detailed time course during the parr–smolt transformation. The results identified five receptor genes and four olfactory‐specific genes that were differentially regulated, supporting olfactory differentiation prior to downstream migration, which may be involved in imprinting and subsequent homing.

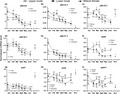

## 1 INTRODUCTION

The anadromous salmonid life cycle begins in small freshwater streams, where eggs are hatched, and the juveniles stay stream‐dwelling at the stage called parr. During the first year, Atlantic salmon typically differentiate into two growth modal groups (Thorpe, 1977) referred to as upper mode (UM) and lower mode (LM). After 1–2 years, UM parr transform into the seawater tolerant smolt stage at the onset of spring, while LM fish remain at the parr stage and require an additional year before smolting at 2+ years (Stefansson, Björnsson, Ebbesson, & McCormick, 2009). At the peak of smoltification, the UM salmon initiate downstream migration and begin a long journey to reach marine feeding areas often thousands of kilometers from home. After 2–3 years at sea, the adults begin a homing migration back to the stream and often the very same spawning bed where they hatched in order to complete the life cycle. Biologists have long been fascinated by the mechanism of homing, and how this can lead to such high degree of precision in navigation. Based on a range of studies including field trials, behavioral experiments, electrophysiological and molecular analyses of olfactory epithelia, and associated neural tissue, there is good evidence that juvenile salmon somehow imprint on the chemistry of their native water (Bett & Hinch, 2016). This information is subsequently used for navigation and recognition upon return (Dittman, Quinn, & Nevitt, 1996; Quinn, 2005; Ueda, Yamamoto, & Hino, 2007). The nature and significance of each scent component are unknown, and a whole cocktail of both biotic and abiotic factors may be involved (Bett & Hinch, 2016).

While at sea, homing salmon navigate by an array of stimuli including magnetic field (Putman et al., 2013), polarized light (Parkyn, Austin, & Hawryshyn, 2003), and scent trails from conspecifics (Nordeng, 1971). When approaching coastal areas and once being in the freshwater system, navigation is based primarily on stream‐specific scents that are picked up by the olfactory sense (Bett & Hinch, 2016). The olfactory system responds to specific scents from the stream including amino acids and kin‐related molecules arising from bile, intestinal content, urine, and skin mucus (Bett & Hinch, 2016). Furthermore, it has been shown that adult salmon respond more intensely when experiencing fragrances to which they have been exposed earlier on in life (Cooper & Hasler, 1974; Dittman, Persons, May, Couture, & Noakes, 2015; Morin, Dodson, & Doré, 1989; Nevitt, Dittman, Quinn, & Moody, 1994; Scholz, Horrall, Cooper, & Hasler, 1976). The available evidence strongly suggests that juvenile salmon, while still in their native stream, imprint on a scent pattern which can be evoked and used for navigation later in life. Functional evidence has shown that the neural sensitivity to specific chemicals such as alanine varies through the parr**–**smolt transformation (PST; Morin & Døving, 1992) and that imprinting to artificial chemicals is most efficient at the smolt stage but also at embryonic stages (Dittman et al., 2015, 1996). Thus, there may be restricted sensitive periods where odor memorization takes place. There is, however, variability related to species differences and experimental conditions.

A major challenge in the study of homing mechanisms is to identify the molecular receptor types involved in imprinting and recognition of odorant cocktails (e.g., pheromones, amino acids, bile salts, prostaglandins; Bett & Hinch, 2016). Odorant perception is based on ligand–receptor interaction and involves membrane‐spanning G protein‐coupled odorant receptors in the olfactory rosette epithelium (Hamdani & Døving, 2007). Three different cell types are present in this epithelium, each cell expressing only one receptor type in a characteristic scheme: ciliated neurons, crypt cells, and microvillous neurons. Four main families of olfactory receptor proteins are expressed in the olfactory epithelium: (a) main odorant receptors (MORs) expressed in ciliated neurons, (b) vomeronasal type 1 receptors (V1Rs known as ORAs) expressed in crypt cells, (c) vomeronasal type 2 receptors (V2Rs known as OlfCs) expressed in microvillous neurons, and (d) trace amine‐associated receptors (known as TAARs) where the cell type is not yet identified (Hino, Miles, Bandoh, & Ueda, 2009). Although the specific ligand types are not fully clarified, the different types of receptors supposedly bind different types of molecules as ligands. As suggested in the references, MORs may use odorants (Wickens, May, & Rand‐Weaver, 2001), ORAs may use pheromones (Ahuja & Korsching, 2014; Saraiva & Korsching, 2007), OlfCs may specifically bind amino acids, and TAARs may use biogenic and trace amine as ligands (Syed et al., 2015; Tessarolo, Tabesh, Nesbitt, & Davidson, 2014). In fish, the main olfactory receptors and the vomeronasal receptors are present in the same epithelium of the nasal cavity, in contrast to terrestrial vertebrates, where olfactory receptors are expressed in the olfactory epithelium, and vomeronasal receptors are expressed in a separate vomeronasal organ. Specific odor recognition in fish then involves a nonspatial patterning of receptor activation in the three types of neurons in combination with convergence of this information to a specific region in the olfactory bulb and subsequent relay to the telencephalon (Hamdani & Døving, 2007).

The number and diversity of olfactory receptor (OR) genes are variable between vertebrates; in fish, it is generally only a fraction of what is known from mammals. While more than 1,000 genes are present in mouse (Zhao & Firestein, 1999), 143 intact OR genes have been identified in the zebrafish genome, yet showing greater sequence diversity than in mammals (Alioto & Ngai, 2005). The first salmonid odorant receptor, named Atlantic salmon odorant receptor (ASOR1) belonging to the MOR family, was characterized by Wickens et al. (2001). Based on the Atlantic salmon Genome Project (Davidson et al., 2010), 24 *mor* genes, seven *ora* genes, 29 *olfc* genes, and 27 *TAAR* genes and a comparable number of putative pseudogenes in each family have subsequently been identified in Atlantic salmon (*Salmo salar*: Johnstone, Lubieniecki, Koop, & Davidson, 2012; Tessarolo et al., 2014). As a logical next step, attempts have been made to establish transcript dynamics for some of these receptors, and comparisons have been made between life stages with the aim to identify a suite of receptors, which may become activated during the PST. Single studies have focused on individual genes in separate species, and as such, there is no clear picture among salmonids. In Atlantic salmon, Johnstone, Lubieniecki, Koop, and Davidson (2011) identified seven potential OlfC receptor (V2R) genes which displayed significantly different expression levels between juveniles and adults but so far no key receptor(s) has shown consistent differences in expression levels between parr and smolts. Prior to that, Dukes, Deaville, Bruford, Youngson, and Jordan (2004) and Dukes et al., (2006), analyzed three receptor genes from Atlantic salmon, which were also identified later by Johnstone et al. (2011): *sorb* = *mor115‐6*, *svra* = *olfc4.9*/pseudogene, and *svrc* = *olfc16.1*. They found some temporal variation in developing smolts which was, however, variable between the two salmon stocks examined. Thus, no firm conclusion could be made on which receptor genes—if any—have the key roles in imprinting, or whether there is a consistent developmental variation in receptor expression during the PST.

In addition to odorant receptors, attempts have been made to identify developmental changes in olfactory system‐related proteins during smolting. Using the GRASP 16k cDNA microarray, Robertson and McCormick (2012) reported 88 features (out of 233 analyzed) in the olfactory rosette that were differentially expressed between parr and smolt. Other studies have taken a more focused approach to analyze individual genes, such as salmon olfactory imprinting‐related gene (SOIG), glutathione‐S‐transferase (GST also named N24), UDP‐glucuronosyltransferase (UGT), and ependymin. SOIG is a member of the Ly‐6 superfamily of proteins (Wang, Dang, Johnson, Selhamer, & Doe, 1995) and has resemblance to urokinase plasminogen activator surface receptor (uPAR), a membrane‐anchored receptor using urokinase as ligand. Its function in the salmon olfactory epithelium is unknown, and however, another member of the Ly‐6 protein family (ODR‐2) has a crucial function for olfaction in *Chaenorhabditis elegans* (Chou, Bargmann, & Sengupta, 2001). SOIG was specifically located in the olfactory epithelium of lacustrine sockeye salmon (*Oncorhynchus nerka*), where it may be associated with neural proliferation during learning (Hino, Iwai, Yamashita, & Ueda, 2007). Accordingly, SOIG mRNA levels surge during PST and during homing migration in lacustrine sockeye salmon (Yamamoto, Hino, & Ueda, 2010). SOIG has not been reported in other salmonid species so far. GST (Kudo et al., 1999) and UGT (Lazard et al., 1991) are detoxification enzymes that may be involved in neuromodulation and in termination of odor signaling by degrading/conjugating odorant molecules (Hino et al., 2009). Ependymin is a neurotrophic factor, which has long been thought of as an effector of long‐term memory consolidation in fish (Bernier, Birkeland, Cipriano, McArthur, & Banks, 2008; Lado et al., 2013). It was upregulated in fall‐run mature chinook salmon (*O. tshawytscha*) compared with spring‐run and ocean‐dwelling fish and was suggested to have a role in memory formation in homing salmon (Bernier et al., 2008). Ependymin has not been analyzed before during the PST of any species. Neurogenin‐1 and neuronal differentiation factor 4 are transcription factors both involved in the embryonal differentiation of neural tissue in the olfactory placode in zebrafish (Madelaine, Garric, & Blader, 2011; Miyasaka et al., 2013), and their dynamics during PST have not been reported. Olfactory marker proteins (OMP1 and OMP2) are specifically expressed in a subpopulation of mature olfactory neurons in *O. nerka* (Kudo, Doi, Ueda, & Kaeriyama, 2009). Their role is unknown and their dynamics have not been reported during the PST.

Thyroid hormones (THs) are fundamentally involved in neurogenesis and neural ontogeny in vertebrates (Campinho, Saraiva, Florindo, & Power, 2014; Kapoor, Fanibunda, Desouza, Guha, & Vaidya, 2015). THs are also essential for growth and maturation of olfactory neurons in rats (Paternostro & Meisami, 1996). In salmon, they are essential regulators of various aspects of the PST, for example, metabolism (Björnsson, Stefansson, & McCormick, 2011), body silvering (e.g., Miwa & Inui, 1985), and initiation of downstream migration (Ojima & Iwata, 2007), and the classical surge in their plasma levels is an innate part of the endocrine profile of the PST (e.g., Dickhoff, Folmar, & Gorbman, 1978; Grau, Dickhoff, Nishioka, Bern, & Folmar, 1981). TH surges are also induced by changes in water chemistry (Hoffnagle & Fivizzani, 1990) and have been proposed to play a significant role in downstream migration and sequential imprinting (Nevitt et al., 1994). Triiodothyronine (T3) has been shown to induce cellular proliferation in the olfactory epithelium of parr, which corresponds to the changes seen in fish undergoing natural smoltification (Lema & Nevitt, 2004), and it has also been shown that T4 administration to chum salmon juveniles stimulates the N‐methyl‐D‐aspartate receptor subunit NR1 mRNA level—which plays an important role in memory formation and retrieval in higher vertebrates and in fish (Ueda et al., 2016).

Based on the available literature, we chose to analyze transcript levels of selected olfactory receptors and olfactory‐related proteins on a 3‐week interval time course from January to June and to compare UM developing Atlantic salmon smolts with LM nonsmolting individuals and wild mature returning females caught in November. With the assumption that imprinting is an integrated feature of smoltification and involves preparatory changes in the olfactory system, we hypothesized that smolting juveniles display temporal, modal as well as life stage‐specific differences in the expression of some of the analyzed olfactory targets. Additional experiments were done to investigate the ability of T3 to differentially regulate the expression of olfactory genes by bolus injection into presmolts in vivo and by direct exposure of isolated olfactory rosettes to T3 ex vivo.

## 2 MATERIALS AND METHODS

### 2.1 Fish and rearing conditions

**Figure 1:**
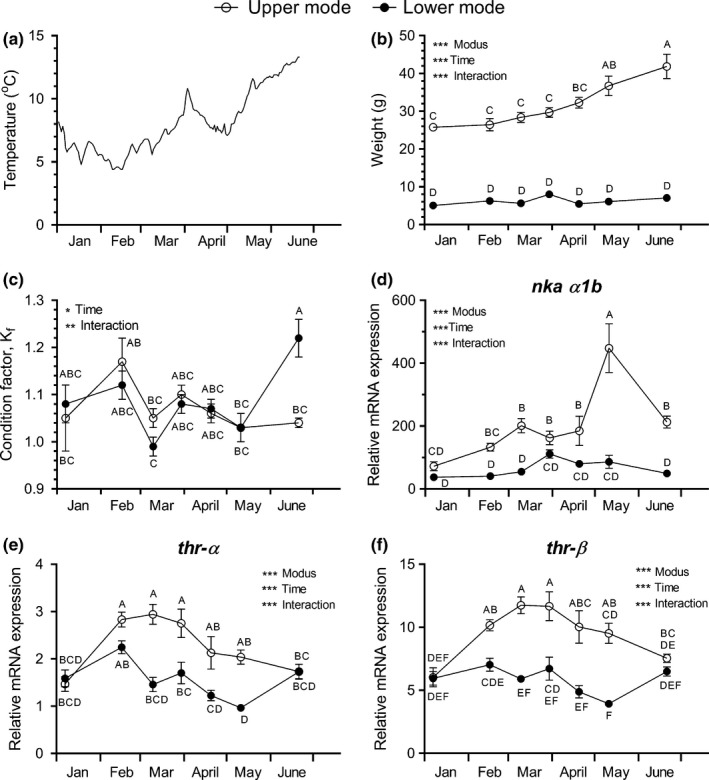
Seasonal changes in water temperature (a), body weight (b), condition factor, *K*_f_ (c), normalized gill Na^+^,K^+^‐ATPase α‐1b subunit (*nka α1b*) transcript level (d), normalized thyroid hormone receptor α (*thr‐α*) (e), and *thr‐β* (f) in the olfactory epithelium of juvenile Atlantic salmon. Upper (o) and lower mode (•) fish were sampled from January to June. Transcript levels were normalized to the geometric mean of *β‐actin* and *ef‐1a* levels. Data in b, c, d, e, and f were analyzed by two‐way ANOVA followed by Tukey's pairwise comparison tests of all means against each other. Shared letters indicate that means were not significantly different (*p* < .05). *, **, *** Indicate significant overall ANOVA effect (*p* < .05, *p* < .01, *p* < .001, respectively) of TIME, MODUS, and INTERACTION as indicated. Data are shown as mean ± *SEM* (*n* = 8)

For the main seasonal smolt experiment, 1‐year‐old Atlantic salmon (Vestjydske strain) was reared from eggs (2016 year‐class) at the Danish Center for Wild Salmon (Randers, Denmark). They were kept indoor under simulated natural photoperiod for latitude of 56°N and temperature (Figure 1a) in bio‐filtered, recirculated freshwater (local well water; tank size: 2.6 m^3^; water change: 0.3–0.5 L/s; and fish density: 50–55 kg/m^3^). Fish were fed commercial salmon pellets ad libitum throughout the study (Aller Performa grade 0–3, Aller Aqua A/S). For comparison with the smolt experiment, nine mature wild Atlantic salmon females (76–97 cm, Vestjydske strain) were caught by electrofishing on a 5 km stretch of the River Gudenaa downstream of the Tange Power Station (Jutland, Denmark) during their homing migration in November and sampled as described below.

All experimental procedures were approved by the Danish Animal Experiments Inspectorate in accordance with the European convention for the protection of vertebrate animals used for experiments and other scientific purposes (#86/609/EØF).

### 2.2 Experiments

#### 2.2.1 Seasonal smolt experiment

In early January 2017, a batch of 1‐year‐old fish was sorted into upper modal (UM; >25 g) and lower modal (LM; <8 g) growth groups (Thorpe, 1977) and kept in separate tanks. Feeding was continued as described above. Sampling of eight fish from UM and LM groups was then performed on January 6, February 15, March 9, March 28, April 19, May 10, and June 20. Upon sampling, fish were anaesthetized in an overdose of bicarbonate‐buffered MS‐222 (tricaine methanesulfonate; Sigma‐Aldrich). After making weight and length measurements to the nearest 0.1 g and 0.1 cm, respectively, the fish was killed by decapitation and brain pithing, one gill arch was dissected and frozen in dry ice, and the snout was cut away posterior to the nasal openings. The snout was then split into two halves each representing one nasal opening and the underlying olfactory rosette, and extraneous cartilage, bone, and skin were trimmed away before putting the tissue in RNA later (Invitrogen) and stored at 4°C. Within 1–3 days, the two olfactory rosettes were dissected free and immediately homogenized in 0.5 ml TRI reagent (Sigma‐Aldrich). Condition factor, *K*_f_, was calculated as 100 × weight/length^3^.

#### 2.2.2 T3 in vivo implant experiment

In order to test the effect of T3 on the expression of selected olfactory receptors and related protein targets, an in vivo injection experiment was performed in late February 2018 using presmolt salmon from the 2017 year‐class (20–25 g). Two groups of 10 fish were lightly anaesthetized in bicarbonate‐buffered MS‐222 and given an intraperitoneal bolus implant with vegetable oil (control) or 5 μg/g T3 (T3‐sodium salt, Sigma‐Aldrich) suspended in vegetable oil, respectively. The use of oil as a vehicle for T3 implants is an effective method to raise plasma thyroid hormone levels in teleosts (Arjona et al., 2011). After 5 days, the olfactory rosettes were sampled from these fish according to the procedure described above.

#### 2.2.3 T3 ex vivo incubation experiment

In late February 2019, an additional experiment was set up to test the effect of T3 ex vivo on olfactory rosettes isolated from presmolt salmon from the 2018 year‐class (25–30 g). Olfactory rosettes from 16 fish (i.e., 32 rosettes) were sampled as described above and preincubated in chilled salmon Ringer's solution (140 mM NaCl, 15 mM NaHCO_3_, 2.5 mM KCl, 1.5 mM CaCl_2_, 1.0 mM KH_2_PO_4_, 0.8 mM MgSO_4_, 10 mM D‐glucose, and 5.0 mM EPPS (4‐(2‐hydroxyethyl)‐1‐piperazinepropanesulfonic acid); equilibrated with 99:1% O_2_:CO_2_, pH 7.8) containing 400 units penicillin, and 400 units streptomycin/ml for 1 hr after the last sampling. Then, the rosettes were randomly assigned to one of the four T3 treatments by distributing each rosette into one well of 24‐well plates containing 1 ml of salmon Ringer's with the addition of one of the following doses of T3: 0, 1, 10, or 100 ng/ml T3 (*n* = 8). The rosettes were incubated with gentle shaking in a 99:1% O_2_:CO_2_ atmosphere at 12 degrees (rearing temperature) for 48 hr, and Ringer's solution being changed after 24 hr. At the end of the incubation period, rosettes were transferred to TRI reagent and immediately homogenized.

### 2.3 RNA extraction, first‐strand cDNA, and real‐time qPCR

Total RNA was extracted following the Tri reagent protocol from the manufacturer. The yield of RNA was between 1 and 4 μg dissolved in nuclease‐free water. The ratio A260/A280 measured on a NanoDrop 1000 (Thermo Fisher Scientific) was 1.9–2.0 indicating high purity RNA. Five hundred nanogram of RNA was used for first‐strand cDNA synthesis using the Applied Biosystems high‐capacity reverse transcription kit (Thermo Fisher Scientific) in a total of 20 μl. Twenty microliter of sterile water was added to the cDNA before running qPCR.

Using Primer3 software (Koressaar & Remm, 2007; Untergrasser et al., 2012), primers for SYBR‐green‐based qPCR were designed as intron spanning where possible, otherwise generated within an exon. Primers were generated to analyze the mRNA level of olfactory receptor genes (MOR‐type: *mor115‐6 (sorb)* and *asor1*; V1R‐type: *ora1* and *ora2*; V2R‐type: *olfc4.9*, *olfc13.1*, *olfc15.1*, *olfc16.1*, and* olfc17.1*), mRNAs encoding olfactory‐related proteins (*gst*, *ugt*, *ependymin*, *soig*, *omp1*, and *omp2*), transcription factors (*neurog1 *and* neurod4*), and thyroid hormone receptors (*thr‐α* and *thr‐β*). Gill Na^+^, K^+^‐ATPase alpha 1b primers (*nka‐α1b*) were used from Madsen, Kiilerich, and Tipsmark (2009). The qPCR protocol (two‐ or three‐step; annealing/elongation temperature) was optimized for each primer, and the primer concentration was 200 nM. Elongation factor‐1a (*ef‐1a*) and beta‐actin (*β‐act*) were used as normalization genes. All information concerning primers, sequences, design, amplicon length, annealing temperature, qPCR protocol, amplification efficiency, and accession number is listed in Table 1.

**Table 1:** Information on *Salmo salar* qPCR targets and related primers used for the analyses

Target (alternative name)	Target type	Primer sequences	Intron spanning^a^	qPCR^b^	*T*_ann_	Ampl. size	*E*_a_	Accession number	Reference	*C*_t_ range
*sorb (mor115‐6)*	*mor*	FP: CTCACCTTCACCATTGTCCTCTT RP: AGCACTCGGCTGCGATCT	−	2	58.2	79	111	XM014138065.1	Dukes et al. (2004) and Johnstone et al. (2012)	21–23
*asor1*	*mor*	FP: CCTGTCTCACCCATCTGCTG RP: ATTGAGCATGGGTGGCAGG	+	3	61.2	146	99	AY007188	Wickens et al. (2001)	23–26
*ora1*	*V1R*	FP: GGGTCTTCTTCTCCTGCTGT RP: CTGGGTGTTGACTGATGGCT	−	3	61.2	125	91	EU143808.2	Johnstone et al. (2008)	21–25
*ora2*	*V1R*	FP: CTGCTGGTGGTAGGTGTGC RP: GATGGAGAGGGAACGAGACG	−	3	58.2	126	90	EU143809.2	Johnstone et al. (2008)	23–26
*olfc4.9 (svra)*	*V2R*	FP: GGCATCAAACGCTCTGTCAT RP: ATCCTCACTGCCATCACACA	+	3	63.8	147	105	HM133620	Dukes et al. (2004) and Johnstone et al. (2012)	18–24
*olfc13.1*	*V2R*	FP: TGTCTGCTGCTTCGACTGC RP: TGGAACACAGTGGTCTCTG	+	3	62.4	124	105	HM133609	Johnstone et al. (2012)	23–27
*olfc15.1*	*V2R*	FP: CCCCAGTGATGTCTACCAGG RP: TACCCCGAATCTCCTCCTCA	+	3	63.8	137	100	HM133612	Johnstone et al. (2012)	21–24
*olfc16.1 (svrc)*	*V2R*	FP: TCAGCAACACCACAAACTCG RP: AACGAGGCAGTCATCAAGGA	+	3	60.0	145	106	HM133613	Dukes et al. (2004) and Johnstone et al. (2012)	20–24
*olfc17.1*	*V2R*	FP: TCAACCTCCACATCCACCAT RP: AGTCACTGGGGATGGTTCTG	+	3	60.0	139	108	HM133605	Johnstone et al. (2012)	20–25
*soig* c * (uPAR)*	ORP	FP: ACACTTTCTCTGACGACCCC RP: ACCGACCATTACTAGCTGTCA	+	3	61.2	140	101	XM014215395.1 XM014215397.1	Hino et al. (2007)	18–25
*ependymin*	OPR	FP: TGATGCCCTTCTGCTCTTCA RP: CCCTCTTCAGCCTCTCCTTC	−	3	60.0	86	97	BT056665.1	Bernier et al. (2008) and Lado et al. (2013)	18–23
*gst (N24)*	ORP	FP: GCCTTCGTTTGCTGACTACA RP: GGACGGGCTGACATCTTCTC	+	3	59.0	120	118	XM014199302.1	Kudo et al. (1999)	15–18
*ugt*	ORP	FP: TCGGACCAAATGACCTTCGG RP: GCTTATCGTAGTGTGGGCTGA	−	3	60.8	100	88	NM001139871.1	Lazard et al. (1991)	22–26
*thr‐α* d	Nuclear receptor	FP: AGGGAGATGAGAAACGGTGG RP: CGGCTCATCCTTCTCCAAGT	+	3	62.2	134	100	NM001123628.1	Marchand et al. (2001)	20–24
*thr‐β* d	Nuclear receptor	FP: TGAAGGGAGCAAAGTGGACA RP: GCAGCTCACAGAACATAGGC	+	3	60.0	140	91	NM001123700.1	Marchand et al. (2001)	20–24
*neurog1*	Transcription factor	FP: CTCCCCGTCATCCTCGATTG RP: GTGGCTTCATTCCTCGCTCT	−	3	62.4	117	94	XM_014198547.1	GenBank	21–26
*neurod4*	Transcription factor	FP: CAGGAGAGGTTCAAGGCGAG RP: AGTTTCTGGGTCTTGGAGGC	−	3	62.4	125	100	XM_014135850.1	GenBank	21–26
*omp1* e	ORP	FP: CCTCACCCACCTGATGAACC RP: ATCCGCTCACCAAACTCCTG	−	3	61.2	126	92	XM_014197736.1 XM_014137085.1	GenBank	15–20
*omp2* e	ORP	FP: CTCTGGACCCCTGACCTCA RP: CTCCAGACACTTGAGGGGC	−	3	61.2	111	92	XM_014214500.1 XM_014162671.1	GenBank	19–23
*ef‐1a*	Housekeeping gene	FP: GAGAACCATTGAGAAGTTCGAGAA RP: GCACCCAGGCATACTTGAAAG	−	2	60.0	71	97	AF321836	GenBank	15–18
*β‐actin*	Housekeeping gene	FP: TGGGACGACATGGAGAAGAT RP: AGAGGCGTACAGGGACAACA	−	3	60.0	201	101	XM014194537.1	GenBank	14–17

Note

Olfactory receptor nomenclature is used according to Johnstone et al. (2008) and Johnstone et al. (2012). Alternative names are listed in brackets.

Abbreviations:* asor*, Atlantic salmon odorant receptor; *E*_a_, amplification efficiency; *ef‐1a*, elongation factor‐1a; *gst*, glutathione sulfonate‐transferase; *mor*, main olfactory receptor; *neurod4*, neurogenic differentiation factor 4; *neurog1*, neurogenin; *omp1/2*, olfactory marker protein 1/2; *ora*, olfactory receptor class A; ORP, olfactory‐related protein; *soig*, salmon olfactory imprinting‐related gene; *sorb*, salmon olfactory receptor class B; *svra/c*, salmon vomeronasal receptor class A/C; *ugt*, UDP‐glucuronosyltransferase; *V1R*, vomeronasal type 1 receptor; *V2R*, vomeronasal type 2 receptor.

a “+” and “−” refer to primers being intron spanning or not, respectively.

b Refers to qPCR protocol as being 2‐ or 3‐step (see Section 2).

c Primers are specific for both transcript variants of the SOIG gene.

d Primers for the thyroid hormone receptors are highly isoform specific.

e Primers are specific for both transcript variants listed; omp nomenclature was based on alignment with GenBank sequences for *Danio rerio omp1* (NM_173281.2) and *omp2* (NM_001025185.1).

Real‐time qPCR was performed using the BioRad CFX96 platform (BioRad) and iTaq Universal SYBR Supermix^®^ in a total volume of 15 μl. The thermocycling protocol consisted of 3‐min initial denaturation (95°C) followed by 40 cycles of either a two‐step protocol (95°C, 15 s; *T*_ann/elong_, 1 min) or a three‐step protocol (95°C, 15 s; *T*_ann_, 15 s; 72°C, 45 s) followed by dissociation curve analysis (65–95°C, 5 s/°C). PCR amplification efficiency, *E*_a_, was analyzed using a 64‐fold dilution range of a pooled cDNA sample, and the relative copy numbers were calculated according to Pfaffl (2001) as follows: $\text{rcn}={\left(1+E_{a}\right)}^{-C_{t}}$, where *C*_t_ is the threshold cycle of the target gene. Corrected rcn data for the two normalization genes were used for calculating their geometric mean and used for normalization of all expression data. The normalization genes were stably expressed in all experiments with no significant effect of any treatment variable. Contamination of RNA samples with genomic DNA was checked for nonintron spanning targets by running QPCR on randomized, diluted RNA samples (“no amplification control”). Amplification in these samples was always <2^−8^ of the corresponding cDNA sample. Primer–dimer association was checked in “no template controls” without addition of cDNA. The molecular mass of all amplicons was validated by gel electrophoresis using a 3% SeaKem Metaphor agarose gel (Lonza), 1 × TAE (40 mM Tris, 20 mM acetic acid, 1 mM EDTA), 5 V/cm, a 20 bp DNA ladder, and 0.4 μg/ml ethidium bromide.

### 2.4 Statistics

The data were tested for outliers using Grubb's test and for normality and homogeneity of variance using Shapiro–Wilk's test and Levene's test, respectively, which necessitated transformation of some of the data using the square root or log(10) functions. In the seasonal experiment, main factorial effects among the juveniles (MODUS: difference between UM and LM groups, and TIME: difference between sampling times) and their interactions were analyzed using a parametric two‐way analysis of variance (ANOVA). ANOVAs showing significant interaction between the two factors (MODUS and TIME) were followed by Tukey's post hoc test to establish pairwise significance of differences between means, including the “Adult” group. In the T3 injection experiment, the two pooled rosettes from each fish were analyzed for mRNA levels related to the 12 target genes. To gain insights into correlated responses among genes and to reveal potential outliers of individual fish in their mRNA levels, we performed a PCA analysis (data shown in Figures S1 and S2). In order to evaluate overall mRNA responses across all genes, we executed a MANOVA. Since mRNA signals were little correlated and not driven by single outliers, we followed these multivariate analyses by pairwise comparisons for each gene, where we performed two‐tailed Student's *t* tests with or without Welch correction for unequal variances as appropriate. In the T3 ex vivo experiment, the mRNA levels of the 12 targets were evaluated in individual rosettes being exposed to different levels of T3. Similar to the in vivo experiments, to investigate correlated responses among the 12 targets, to reveal potential outliers that would weigh heavily on findings, and to quantify overall response to the treatment, we first performed multivariate level analysis (PCA and MANOVA), followed by individual gene level analyses for which we used a one‐way ANOVA. These ANOVAs were then followed by pairwise comparisons between the control group and each treatment group separately using Dunnett's test. Differences between means were accepted as statistically significant at *p* < .05. All multivariate statistical procedures were made using R (R Core Team, 2017; using packages factoextra for plotting), while the single response analyses were made using Prism 8.1 (GraphPad Software).

## 3 RESULTS

### 3.1 Smoltification indices

Water temperature in the rearing tanks increased steadily from 5–6°C in January to a maximum of 13°C in June (Figure 1a). The two modal groups had distinctly different body weights with lower mode fish averaging 6–8 g and upper mode fish increasing from about 26 g in January to roughly 40 g in June (Figure 1b). Condition factor, *K*_f_, fluctuated between samplings and showed no clear developmental trend nor difference between UM or LM groups (Figure 1c). Gill Na^+^, K^+^‐ATPase alpha 1b (*nka‐α1b*) RNA levels were low and stable in the LM group, consistently higher in the UM group (except January) and furthermore showed an increase with a distinct peak in May, followed by a steep decline in June (Figure 1d). Thyroid receptor alpha (*thrα*) and beta isoform (*thrβ*) transcript levels displayed highly significant effects of MODUS, TIME, and their interaction and were elevated in the UM groups from February to May compared with the LM groups (Figure 1e,f). Visual appearance also developed distinctly different in the two groups. LM fish showed typical parr appearance with parr marks along their sides throughout the sampling period. UM fish gradually developed an intense silvery appearance typical of smolts with darkening of fin edges and loosening of scales reaching a climax in May. There were no signs of precocious (male) maturity in any of the sampled fish.

### 3.2 Olfactory receptor gene expression during PST

**Figure 2:**
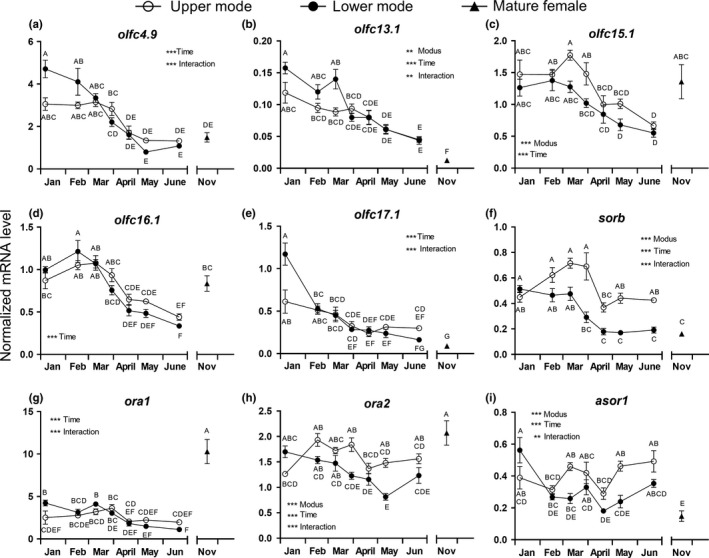
Seasonal variation in normalized transcript levels of olfactory receptors in the olfactory epithelium of juvenile Atlantic salmon. Upper (o) and lower mode (•) fish were analyzed at multiple time points and compared with mature females (Δ) caught in November in the river Gudenaa. (a) *olfc4.9*, (b) *olfc13.1*, (c) *olfc15.1*, (d) *olfc16.1*, (e) *olfc17.1*, (f) *sorb*, (g) *ora1*, (h) *ora2*, and (i) *asor1*. Transcript levels were normalized to the geometric mean of *β‐actin* and *ef‐1a* levels. Data were analyzed by two‐way ANOVA followed by Tukey's pairwise comparison tests of all means against each other. Shared letters indicate means that were not significantly different (*p* < .05). *, **, *** Indicate significant overall effect (*p* < .05, *p* < .01, *p* < .001, respectively) of TIME, MODUS, and INTERACTION. Data are shown as mean ± *SEM* (*n* = 8). Note that the *y*‐axes are differently scaled

The *C*_t_ values were generally in the range 13.2–17.2 for the normalization genes and 15.1–28.3 for the target genes (see details in Table 1). There was a significant effect of TIME as treatment variable on the mRNA level of all receptor genes (Figure 2). Five genes showed a significant effect of MODUS (LM vs. UM; *olfc13.1*, *olfc15.1*, *sorb*, *ora2*, and *asor1*), while in seven genes there was a significant interaction between TIME and MODUS (*olfc4.9*, *olfc13.1*, *olfc17.1*, *sorb*, *ora1*, *ora2*, and *asor1*). There were specific patterns of variation in each gene with respect to TIME and MODUS. The most pronounced modal difference between UM and LM was observed with regard to *olfc15.1*, *sorb*, *ora2*, and *asor1*, which all displayed higher expression in the UM than in the LM group during most of the study period. In addition, there was a fairly consistent downward trend for several of the target mRNAs in both UM and LM groups during the study.

### 3.3 Olfactory‐related proteins during PST

**Figure 3:**
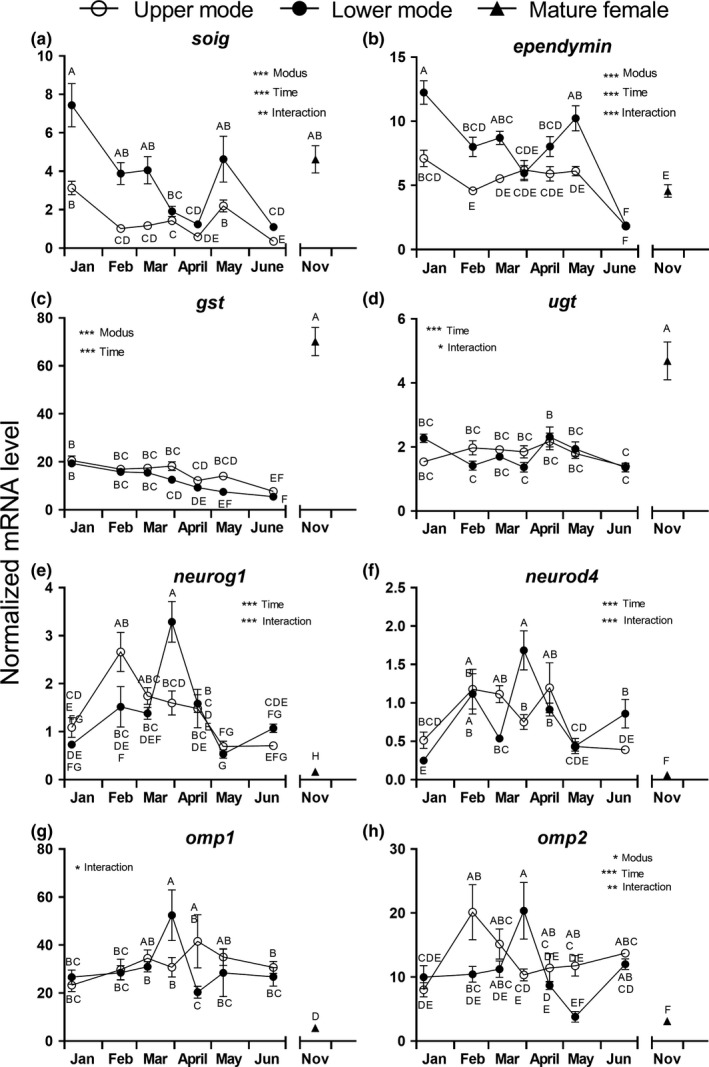
Seasonal variation in normalized transcript levels of olfactory system‐specific proteins in the olfactory epithelium of juvenile Atlantic salmon. Upper (o) and lower mode (•) fish were analyzed at multiple time points and compared with mature females (Δ) caught in November in the river Gudenaa. (a) *soig*, (b) *ependymin*, (c) *gst*, (d) *ugt*, (e) *neurog1*, (f) *neurod4*, (g) *omp1*, and (h) *omp2*. Transcript levels were normalized to the geometric mean of *β‐actin* and *ef‐1a* levels. Data were analyzed by two‐way ANOVA followed by Tukey's pairwise comparison tests of all means against each other. Shared letters indicate means that were not significantly different (*p* < .05). *, **, *** Indicate significant overall effect (*p* < .05, *p* < .01, *p* < .001, respectively) of TIME, MODUS, and INTERACTION. Data are shown as mean ± *SEM* (*n* = 8). Note that the *y*‐axes are differently scaled

The mRNA levels of all olfactory‐related proteins except *omp1* were significantly affected by TIME (Figure 3). In four genes, there was a significant effect of MODUS (*soig*, *ependymin*, *gst*, and* omp2*), and in seven genes, there was a significant interaction between TIME and MODUS (*soig*, *ependymin*, *ugt*, *neurog1*, *neurod4*, *omp1*, and* omp2*). *gst* and *omp2* had higher expression in UM fish than LM fish at the peak of smoltification in May, whereas *soig* and *ependymin* were higher in LM fish through most of the sampling season. In LM fish, *neurog1*, *neurod4*, and the two *omp* genes showed a distinct peak late March compared with LM groups before and after this sampling. *omp2* was significantly elevated in UM fish from January to February and stayed elevated until June.

### 3.4 Comparison of homing mature females with juveniles

The mRNA levels of *ora1*, *gst*, and *ugt* (Figures 2g and 3c,d) were significantly higher in mature females than in juvenile UM and LM fish at any point in time. On the other hand, *olfc13.1*, *olfc17.1*, *sorb*, *asor1*, *neurog1*, *neurod4*, *omp1*, and *omp2* mRNA levels were significantly lower in adults than in any of the UM and most of the LM groups (Figure 2b,e,f,i and 3e,f,g,h). All other olfactory receptors or olfactory‐related proteins had transcript levels in mature females similar to the range seen in juveniles during the sampling period.

### 3.5 T3 experiments

**Figure 4:**
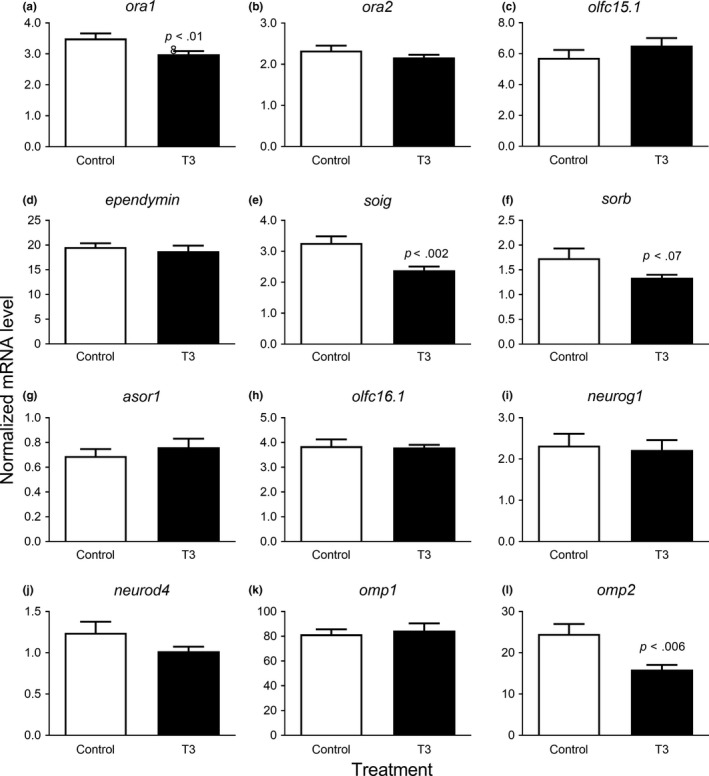
Effect of triiodothyronine in vivo on normalized transcript levels of genes expressed in the olfactory epithelium of presmolt Atlantic salmon. (a) *ora1*, (b) *ora2*, (c) *olfc15.1*, (d) *olfc16.1*, (e) *asor1*, (f) *sorb*, (g) *soig*, (h) *ependymin*, (i) *neurog1*, (j) *neurod4*, (k) *omp1*, and (l) *omp2*. Open bars: sham‐injected control; filled bars: T3‐injected. Data were analyzed by a two‐tailed Student's *t* test with or without Welch correction for unequal variances as appropriate. Significance is indicated above bars. Data are shown as mean ± *SEM* (*n* = 10). Note that the *y*‐axes are differently scaled

**Figure 5:**
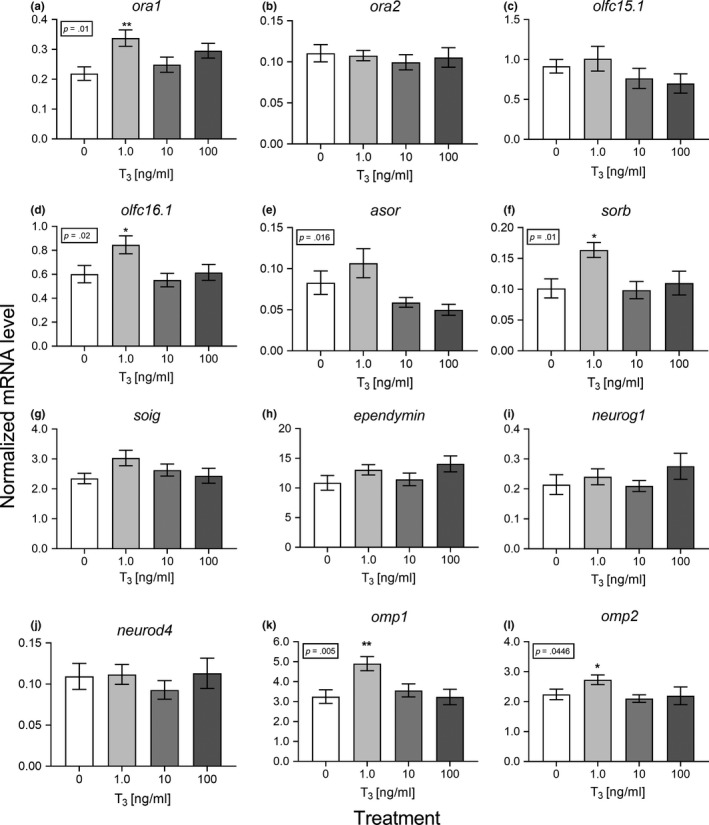
Effect of triiodothyronine ex vivo on normalized transcript levels of genes expressed in the olfactory epithelium of presmolt Atlantic salmon. (a) *ora1*, (b) *ora2*, (c) *olfc15.1*, (d) *olfc16.1*, (e) *asor1*, (f) *sorb*, (g) *soig*, (h) *ependymin*, (i) *neurog1*, (j) *neurod4*, (k) *omp1*, and (l) *omp2*. Open bars: sham‐injected control; filled bars: T3‐injected. Data were analyzed by a one‐way ANOVA (significance is indicated in the boxes) followed by Dunnett's pairwise comparison between the control group and each treatment group. * Indicates *p* < .05. Data are shown as mean ± *SEM* (*n* = 8). Note that the *y*‐axes are differently scaled

Eight targets with modal effects in the main experiment (*ora1*, *ora2*, *olfc15.1*, *olfc16.1*, *sorb*, *asor1*, *ependymin*, and *soig*) together with *neurog1*, *neurod4*, *omp1*, and *omp2* were evaluated with respect to the effect of T3 in vivo and ex vivo. T3 generally had relatively little effect on the selected targets. In vivo, a PCA analysis on the twelve targets combined revealed that the targets were overall weakly correlated and that targets could not be collapsed without losing substantial information (the first two principal components combined explained less than 50% of the total variance, see Figure S1A). These findings, in combination with the findings from the PCA that none of the individuals having exceptional high weights and that individuals did not cluster in an obvious way (Figure S1B), conclude that target effects were largely independent (Figure S1C). Performing an MANOVA on all 12 targets showed a marginal significant effect of T3 (*F*_1,18_ = 3.51; *p* = .052). Due to the weakly correlated target responses (Figure S1) and the marginal significant results of the MANOVA, we decided to present as well single target results. The expression levels of *ora1*, *soig*, and *omp2* were significantly reduced by T3 and *sorb* tended to be reduced by T3 (*p* < .07; Figure 4). In the ex vivo incubation experiment, a PCA analysis on the twelve targets combined revealed that the targets, again as in the in vivo experiments, were not highly correlated (the first two principal components combined explained 62.6% of the variance, Figure S2A). None of the individuals had extreme weights on the PCA or were there any cluster of individuals that shared similar overall characteristics (Figure S2B). None of the targets dominated the PCA but all targets loaded positively on the first component (Figure S2C). Note that the loadings and weights differ substantially among the in vivo and ex vivo experiments. The MANOVA on the ex vivo data combining all 12 targets showed again a tendency of an overall treatment effect (*F*_1,27_ = 2.20; *p* = .071). Due to the indication that target responses among the 12 targets are not well correlated (Figure S2) and the tendency of an overall T3 effect as indicated by the MANOVA, we present also single target results. The one‐way ANOVAs revealed an overall treatment effect on *ora1* (*p* = .01), *olfc16.1* (*p* = .02), *asor* (*p* = .016), *sorb* (*p* = .01), *omp1* (*p* = .005), and *omp2* (*p* = .04). All other targets remained unaffected by T3 (Figure 5).

## 4 DISCUSSION

### 4.1 Seasonal and developmental changes in olfactory gene expression

Wisby and Hasler (1954) originally proposed that anadromous salmon imprint on the water chemistry of their natal stream prior to ocean migration. The precise time period during which imprinting takes place has not been established, yet there is evidence that there may be critical periods both during embryonic life and the PST (Cooper, Scholz, Horrall, Hasler, & Madison, 1976; Dittman et al., 2015; Scholz et al., 1976). This study is the first to investigate olfactory gene expression on a detailed time course during the complete PST and to test the hypothesis that there are developmental changes in gene expression in the olfactory epithelium (OE) which are specific to UM fish during the PST, and thus not present in LM siblings of the same age‐class. The UM fish in our study displayed clear signs of smoltification (elevated thyroid hormone receptor expression in February–April, progressing silvery appearance, fin edge darkening, and a distinct surge in gill *nka‐α1b* mRNA in May), whereas LM fish retained a parr‐like appearance and no change in gill *nka‐α1b*.

We expected changes in odorant receptor expression and signs of neural development in the OE, which may lead to increased sensitivity and ability to recognize significant odors at later life stages. All investigated targets were expressed at relatively high levels in the OE (Table 1), and several genes were differentially expressed between modal groups as well as over time. Modal differences between UM and LM were found in two main olfactory receptors (MOR: *sorb* and *asor1*), one vomeronasal class‐1 (VR‐1: *ora2*) and two class‐2 receptors (VR‐2: *olfc13.1*, *olfc 15.1*), and in four olfactory tissue‐specific proteins *soig*, *ependymin*, *gst*, and *omp2*. *Olfc15.1*, *sorb*, *ora2*, *asor1*, and *gst* were generally expressed at higher levels in UM fish than in LM fish, whereas the opposite was seen in *soig* and *ependymin* expression. *omp2* showed a more complex pattern with a peak in UM fish in February and a peak in late March in the LM fish. Overall, this suggests that different developmental processes occur in the olfactory system in the two modal groups, even though they are same age, reared in the same water, and were exposed to the same odorant cocktail. We conclude that significant changes develop in the olfactory system in relation to the PST, which may lead to increased perception of certain odorants during that period. In addition, seasonal differences (TIME effects) were seen within both modal groups in all genes analyzed except *omp1* and may be related to the change in water temperature during the experimental period.

Previous studies have investigated either single gene targets (Yamamoto et al., 2010) or groups of olfactory targets (Dukes et al., 2004; Johnstone et al., 2011) but at more discrete time points or stages during the PST in UM fish only. Dukes et al. (2004) first reported PST‐related changes in odorant receptor expression in offspring of wild juvenile Atlantic salmon reared in a hatchery environment using water directly from the river. They reported significantly elevated mRNA levels of one MOR receptor (*sorb*) and one V2R receptor (*svra*; similar to *olfc4.9*) in April and June, respectively. Another V2R receptor (*svrc*; similar to *olfc16.1*) showed a nonsignificant elevation in June. Only potential smolts (UM size group) were used in their study, and it cannot be concluded whether the changes are seasonal or developmental. Furthermore, the changes were only seen in one out of two salmon families from the same river, suggesting that increased receptor expression is strongly influenced by a genetic component and may occur multiple times during the spring. The latter observation complies with the sequential imprinting hypothesis proposed by Harden Jones (1968), which implies that smolts are imprinted by sequential odor perception during downstream migration. Our data showed that some of the olfactory receptors displaying higher levels in UM compared with LM fish were mostly elevated during the early phase of the PST in March and in some cases were followed by an abrupt decline in April and onwards (*sorb*, *ora2*, *olfc15.1*, *olfc16.1*, and *omp2*) or continuously elevated through June (*asor1*).

In another major investigation, Johnstone (2011) and Johnstone et al. (2011) analyzed a large suite of receptor genes in three discrete life stages (parr, smolt, and adult) in two anadromous populations of wild‐caught Atlantic salmon. Unfortunately for a comparison with the present study, the precise criteria for classification of the three life stages were not described nor were the times of sampling. It is unknown whether they used LM fish as parr and UM as smolts and what the stage of maturity was in the adults. They did not find any differences between parr and smolt but identified seven *olfc* genes that were consistently downregulated in adults compared with juveniles in both populations (see below). In addition, they reported mRNA levels of *ora1* and *asor* but did not find differences between parr and smolt. Thus, regarding odorant receptor expression during PST, there is little consistency between the few studies available which may reflect the influence of a genetic component, differences between water chemistry, rearing, and sampling conditions. The salmon in the present study were reared in a recycled hatchery environment using well water, Dukes et al. (2004) used natural river water in a hatchery environment, and Johnstone et al. (2011) used wild‐caught fish. Furthermore, different stocks are locally adapted to their environment (Fraser, Weir, Bernatchez, Hansen, & Taylor, 2011) and it is well known that the timing of PST and subsequent seaward migration is genetically variable in Atlantic salmon (Nielsen, Holdensgaard, Petersen, Björnsson, & Madsen, 2001). It should also be kept in mind that the relative stability of water chemistry in hatchery environments may lead to underestimating the imprinting dynamics compared with wild populations where novel water chemistry may be a stimulus per se. There may be an endogenous rhythm in olfactory system development synchronized with the PST and mediated by its endocrine regulators, but exposure to seasonal changes in water chemistry and during migration may be equally important for memorizing the full palette of odorants. A lack of change in water chemistry may put limits on the dynamics of thyroid hormones which regulate major aspects of the PST and may be important for stimulation of olfactory development (Bett & Hinch, 2016; Hoffnagle & Fivizzani, 1990).

We observed a distinct peak in the *soig* level in May in UM fish, but the level was generally higher in LM fish through the whole season. Yamamoto et al. (2010) first reported an increase in *soig* expression during the PST in 1‐year‐old lacustrine sockeye salmon but made no comparison with LM fish. The precise function of the SOIG protein is unknown. It is specifically expressed in the olfactory rosette (Hino et al., 2007), and it is likely that SOIG may have a general role in neural signaling related to olfaction. Another olfactory‐specific protein, GST, was identified in sockeye salmon olfactory receptor neurons by Kudo et al. (1999). The expression of *gst* was generally highest in UM fish and especially during peak smoltification in May. This suggests that GST activity is higher during the period of expected imprinting, which could translate into higher turnover of ligand–receptor interaction. UDP‐glucuronosyltransferase (UGT) is normally associated with detoxification processes in the liver and kidney but is also present in the olfactory system of rats, where it is involved in termination of odorant–receptor interaction (Lazard et al., 1991; Leclerc et al., 2002). We analyzed for the first time expression of *ugt* in the olfactory rosette of salmon, which showed only minor fluctuations over time and similar expression in LM and UM fish. However, *ugt* and *gst* levels were much higher in homing mature females, which signifies more activity in the olfactory system at that stage.

*Ependymin* levels were relatively stable in UM fish from January to May but then dropped significantly in June, when migration normally begins. Lower mode fish, however, had higher *ependymin* mRNA levels through most of the season except for a similar sharp drop in June. Ependymin is generally secreted into the cerebrospinal fluid of the vertebrate brain but is also expressed peripherally in the olfactory epithelium of salmon (Palstra et al., 2015). Memory consolidation was obstructed by intracerebral injection of ependymin antibodies into trained zebrafish (Pradel, Schachner, & Schmidt, 1999), and evidence from goldfish suggests that the ependymin level in the brain decreases transiently during a learning process and is followed by subsequent de novo synthesis (Shashoua, 1991). *Neurog1*, *neurod4*, and *omp2* levels have never been reported in smolting salmon. They all showed a decline around April–May in both UM and LM fish, which together with *ependymin* data suggests that important neuromodulatory events in the olfactory system may take place early in the season independent of the PST.

### 4.2 Olfactory receptor expression in adults during their homing migration

When comparing mature females with smolting juveniles, the most remarkable differences were the considerably elevated levels of *gst* and *ugt* in the adults—two genes involved in termination of odorant signaling as discussed above. The level of *soig* was also higher in adults compared with smolting juveniles but not compared with LM fish. Only one out of the whole suite of receptors analyzed, *ora1*, was expressed at much higher levels in the adult individuals, whereas *olfc13.1* and *olfc17.1*, *sorb*, and *asor1* were expressed at lower levels in adults compared with smolting individuals. Thus, the olfactory gene expression profile in the adult is clearly different from that of juvenile, smolting individuals. This is not surprising since mature adults are in a phase of their homing migration, where they are exposed to a variety of new odors and where increased activity of the olfactory system, and refreshing of the memory is expected. A bias to our study, however, is that juvenile fish were not reared in the same water source as the homing adults were exposed to. It may well be that the suite of olfactory genes that are activated during PST and homing, respectively, is not universal but to a large degree depends on the specific odor cocktail that the fish is exposed to. Thus, it should be expected that there are differences due to year–year, population, and water chemistry effects. Johnstone et al. (2011) identified seven *olfc* genes out of 30 analyzed (*olfc2.2*, *‐3.1*, *‐4.9*, *‐13.1*, *‐15.1*, *‐16.1*, and* ‐17.1*) that were differentially expressed (at lower levels) in returning adults compared with juveniles (parr and smolt) in two populations of anadromous Atlantic salmon. In one of the two populations, 10 additional genes were also expressed at lower levels in adults compared with juveniles. Furthermore, they analyzed *soig* mRNA levels but found no difference between life stages. Palstra et al. (2015) compared by RNAseq expression profiles of 75 known and 27 unknown olfactory genes in coastal adult chum salmon with prespawning individuals caught 75 km upstream in the river. Seven MOR genes, *n24*, *asor*, and two *ependymin*‐*like* genes were significantly upregulated (1.5–2.5×), and *olfc13.1* and *O51F2 HUMAN*—a novel salmonid gene—were downregulated (0.7–0.5×) in prespawning individuals. Bernier et al. (2008) also found elevated *ependymin* levels in the brains of returning adult Chinook salmon. Our data confirm the downregulation of *olfc13.1* and *asor1* in adults, whereas *ependymin* levels were similar in adults and juveniles.

### 4.3 Hormonal regulation of olfactory receptors

It is plausible to speculate that the brain–pituitary–thyroid axis is essential for regulating gene expression in the olfactory epithelium, which forms the basis for imprinting. Kudo, Eto, Abe, and Mochida (2018) showed for the first time expression of thyroid receptor β (*thrβ*) but not *thrα* in the olfactory epithelium of juvenile *O. keta* and our study is the first to report seasonal changes in both TH receptor variants in smolting and nonsmolting salmon. Interestingly, transcript levels of both variants increased in February–April but only in the UM group, which suggests increased sensitivity to thyroid hormones at early stages of smoltification. Thus, our T3 experiments were done with salmon at the presmolt stage. We did not measure plasma T3 levels but the T3 dose used for injection is suspected to induce major elevation in circulating T3 based on a similar protocol used by Arjona et al. (2011).

T3 in vivo and ex vivo failed to induce changes in olfactory gene expression that were similar to those observed during the PST. The targets which were affected by T3 in vivo were *ora1*, *soig*, *sorb* (*p* = .07), and *omp2*, which were all reduced by T3 compared with controls. The negative effect on *soig *in vivo corresponds to the strong modal difference observed between UM and LM fish. The effect on *sorb* does not explain the modal difference in the main experiment but may contribute to the decline in expression level in the UM group in April. The effect on *omp2* is hard to relate to the observed dynamics in the main experiment. Remarkably, these effects were not reproduced in the ex vivo experiment, suggesting that T3 may act indirectly via other hormone interactions. Ex vivo six targets were significantly stimulated by T3 (*ora1*, *olfc16.1*, *asor*, *sorb*, *omp1*, and *omp2*), yet without any dose relationship, with only the lowest dose (1 ng/ml) inducing these effects. We cannot exclude that T3 has a more potent effect on olfactory receptor expression at other stages during the PST. To our knowledge, the effect of thyroid hormone on fish olfactory receptor expression has not been reported previously, and there are no data to compare with. In rats, thyroid hormones are essential for growth and maturation of olfactory receptor neurons (Paternostro & Meisami, 1996) and thyroid hormone replacement improves olfaction and taste sensitivity in hypothyroid patients (Deniz et al., 2016). In our study, *ependymin*, *neurog1*, and *neurod4* markers of neuromodulation were unaffected by T3. T3 is generally assumed to be the active form of thyroid hormone but it should be tested whether thyroxine (T4) has an effect since the olfactory epithelium has deiodinase activity and could potentially convert T4 into T3 locally in the tissue (Plate et al., 2002).

## 5 CONCLUSION

Olfaction is a complex process involving many steps starting with the specific interaction between a ligand and a receptor. The complexity is not least due to the involvement of a large spectrum of receptor variants. It has been estimated that up to 4% of the genome is devoted to encoding receptors and olfactory‐related proteins in higher vertebrates (Firestein, 2001). We analyzed a small subset of known olfactory proteins in Atlantic salmon, and the expression of most of these shows seasonal as well as developmental variation related to life stage (maturity and the parr**–**smolt transformation). Thus, the study supports the hypothesis that certain aspects of olfaction are developed during the PST. T3 did not have any major impact on the expression of any of the targets investigated, and future studies should investigate developmental changes in the sensitivity to both T3 and T4.

## CONFLICT OF INTEREST

None declared.

## AUTHOR CONTRIBUTIONS

SSM conceived and designed the study and wrote the manuscript; SSTW and RJB collected the samples, performed the analyses, and proof‐read the manuscript; US analyzed the data; MHL sampled wild salmon and proof‐read the manuscript.

## Data Availability

Data are accessible at https://doi.org/10.5061/dryad.r7sqv9s7j
