# Supplementary material for: Differential expression of olfactory genes in Atlantic salmon (Salmo salar) during the parr–smolt transformation
Source: Ecol Evol. 2019 Nov 28;9(24):14085–100. doi: 10.1002/ece3.5845 (PMC6953650; doi:10.1002/ece3.5845)
Supplement: Supplementary file 1 [file ECE3-9-14085-s001.docx]

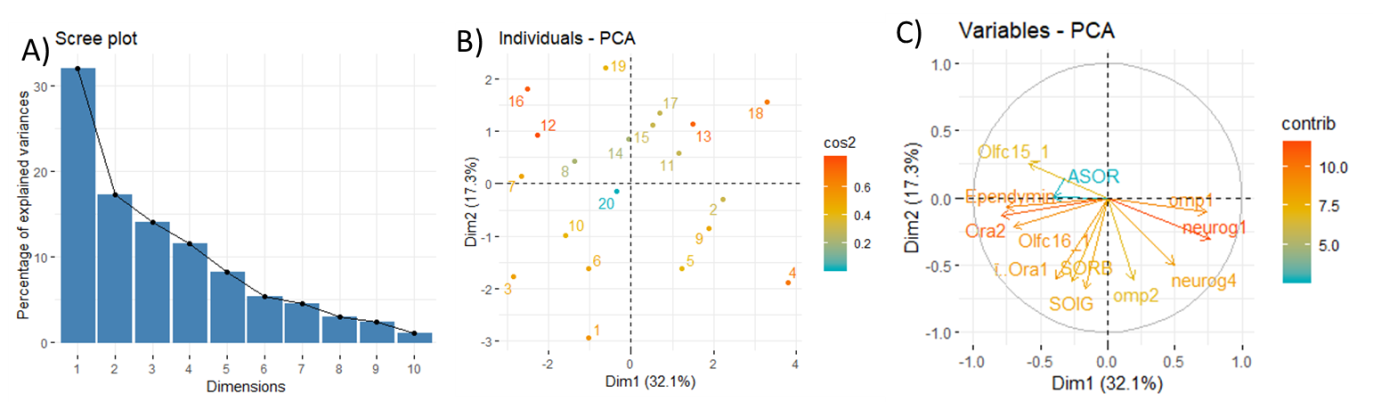


**Figure S1: Principal component analysis of the T3 *in vivo* experiment.** A) Percentage of variance explained by the different Principal components. B) Contributions to the weights for each of the 20 individuals (each data point represents one individual) along the two most important Principal components (red indicates higher weights, blue indicates lower weights). C) Loadings of the 12 different target variables on the two main Principal components (red indicates higher loadings, blue indicates lower loadings).


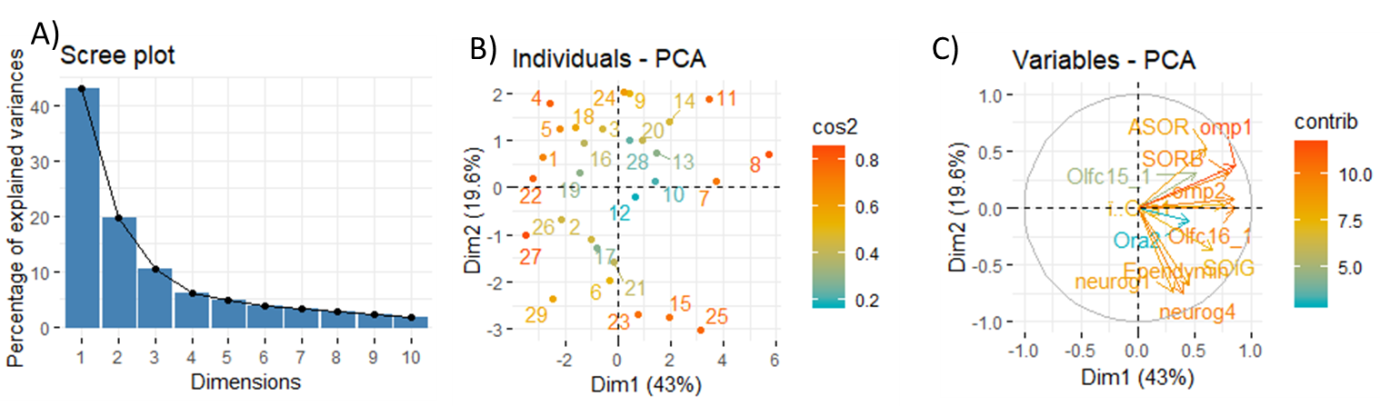


**Figure S2: Principal component analysis of the T3 *ex vivo* experiment.** A) Percentage of variance explained by the different Principal components. B) Contributions to the weights for each of the 29 individuals (each data point represents one individual) along the two most important Principal components (red indicates higher weights, blue indicates lower weights). C) Loadings of the 12 different target variables on the two main Principal components (red indicates higher loadings, blue indicates lower loadings).
